# Electrical Stimulation Therapy and HA/TCP Composite Scaffolds Modulate the Wnt Pathways in Bone Regeneration of Critical-Sized Defects

**DOI:** 10.3390/bioengineering10010075

**Published:** 2023-01-06

**Authors:** Júlia Venturini Helaehil, Luiza Venturini Helaehil, Laryssa Fernanda Alves, Boyang Huang, Milton Santamaria-Jr, Paulo Bartolo, Guilherme Ferreira Caetano

**Affiliations:** 1 Graduate Program in Biomedical Sciences, University Center of Hermínio Ometto Foundation, FHO, Araras 13607-339, Brazil; 2 Singapore Centre for 3D Printing, School of Mechanical and Aerospace Engineering, Nanyang Technological University, Singapore 639798, Singapore; 3 Graduate Program of Orthodontics, University Center of Hermínio Ometto Foundation, FHO, Araras 13607-339, Brazil; 4 Division of Dermatology, Department of Internal Medicine, Ribeirao Preto Medical School, University of Sao Paulo, Sao Paulo 05508-060, Brazil

**Keywords:** bioprinting, electrical stimulation, microcurrent, mineralization, osteogenesis, tissue engineering

## Abstract

Critical bone defects are the most difficult challenges in the area of tissue repair. Polycaprolactone (PCL) scaffolds, associated with hydroxyapatite (HA) and tricalcium phosphate (TCP), are reported to have an enhanced bioactivity. Moreover, the use of electrical stimulation (ES) has overcome the lack of bioelectricity at the bone defect site and compensated the endogenous electrical signals. Such treatments could modulate cells and tissue signaling pathways. However, there is no study investigating the effects of ES and bioceramic composite scaffolds on bone tissue formation, particularly in the view of cell signaling pathway. This study aims to investigate the application of HA/TCP composite scaffolds and ES and their effects on the Wingless-related integration site (Wnt) pathway in critical bone repair. Critical bone defects (25 mm^2^) were performed in rats, which were divided into four groups: PCL, PCL + ES, HA/TCP and HA/TCP + ES. The scaffolds were grafted at the defect site and applied with the ES application twice a week using 10 µA of current for 5 min. Bone samples were collected for histomorphometry, immunohistochemistry and molecular analysis. At the Wnt canonical pathway, HA/TCP and HA/TCP + ES groups showed higher *Wnt1* and *β-catenin* gene expression levels, especially HA/TCP. Moreover, HA/TCP + ES presented higher *Runx2*, *Osterix* and *Bmp-2* levels. At the Wnt non-canonical pathway, HA/TCP group showed higher voltage-gated calcium channel (*Vgcc*), calmodulin-dependent protein kinase II, and *Wnt5a* genes expression, while HA/TCP + ES presented higher protein expression of VGCC and calmodulin (CaM) at the same period. The decrease in *sclerostin* and *osteopontin* genes expressions and the lower bone sialoprotein II in the HA/TCP + ES group may be related to the early bone remodeling. This study shows that the use of ES modulated the Wnt pathways and accelerated the osteogenesis with improved tissue maturation.

## 1. Introduction

Bone tissue is considered as a dynamic tissue maintaining a balance between bone formation and remodeling. However, large-scale defects can directly affect this process [1,2]. Critical defects, associated to large bone loss, requires invasive interventions such as the transplants/grafts. However, both autologous and allogeneic grafts have clinical limitations such as the need of secondary surgery, anatomical limitations, and risk of infection and immune rejection [3].

The production of porous three-dimensional biomaterials (scaffolds) emerged as an alternative to treat bone fractures or bone loss, since scaffolds would act as a support for the cell influx and organized cell growth. The use of biomaterials with the addition of ceramic components, similar to those of natural bone, such as calcium and phosphate (e.g., hydroxyapatite (HA) and tricalcium phosphate (TCP)), has been investigated [2,4,5].

HA is widely used in different medical applications, since it is the main inorganic component of bone and characterized by its osteoconductive potential [6,7]. On the other hand, TCP helps in the proliferation and differentiation of mesenchymal stem cells (MSCs) due to its osteoinductive potential [8,9]. However, due to the poor strength of HA, and high brittleness at high concentrations of TCP, these materials are suggested to be associated with polymers such as polycaprolactone (PCL) to improve their mechanical properties. Moreover, PCL presents adequate biocompatibility and structural and mechanical stability but exhibits low bioactivity. Therefore, the addition of HA and TCP improves its biological performance allowing to obtain composite scaffolds with improved properties [8,10,11,12].

The hydroxyapatite and collagen fibers promote a piezoelectric effect on the bone tissue. The hydroxyapatite controls the amount of water that collagen can absorb to maintain the fiber orientation. In critical-sized defects, the hydroxyapatite is drastically reduced, and the electrical potential significantly decreases on the bone [13]. Therefore, to assist the bone repair process, the application of noninvasive stimulation, such as physical stimulation, could promote bone healing and restore the piezoelectrical potential [14]. 

The physical stimulation could be classified into photonic, mechanical, electromagnetic and electrical. Photonic therapy, like low-level laser therapy (LLLT), could stimulate biochemical reactions that increase mitochondrial activity and the mechanical stimuli promote cell proliferation, and differentiation and enhance cell metabolism. Electrical currents such as electromagnetic and electrical stimulation, at physiological levels, can modulate cellular and molecular signaling pathways enhancing osteogenesis. Electromagnetic stimulation enhances the metabolic activity, cell proliferation and differentiation [13,14,15].

However, it is reported that electrical stimulation has better results on bone repair. At physiological levels, it promotes disorganized bone formation at the beginning of the stimulus application, but throughout the process, the bone becomes more organized, presenting enhanced mineralization rate. These stimuli act on the synthesis of cytokines and growth factors, which are able to enhance angiogenesis, especially mineralization. This therapy activates voltage-dependent calcium channels and Ca^2+^/CaM pathway and modulates signaling pathways such as the wingless-related integration site (Wnt) pathway [14,15,16,17]. The Wnt pathway participates in tissue growth and maintains bone homeostasis. It mainly controls the differentiation of osteoprogenitor cells into osteoblasts [15,16]. Moreover, it is known that Wnt ligands regulate bone healing and consolidation, presenting a great therapeutic potential in the repair of fractures [17]. Three different Wnt pathways were described: the canonical pathway, also known as the Wnt/β-catenin pathway and two non-canonical pathways, the Wnt/Ca^2+^ pathway and the Wnt/PCP pathway. These three pathways are initiated by the binding of extracellular Wnt to the Frizzled receptor (FZD). Depending on the pathway, there is the participation of co-receptors to activate the signal transduction cascade [18]. The Wnt/β-catenin pathway is the most studied, especially for the repair process.

However, the literature has not yet reported the effects of the association between the use of scaffolds and electrical stimulation, and the effects on signaling pathways, such as the canonical and non-canonical pathways. The use of PCL associated with β-TCP 20 wt% and PCL associated with HA 20 wt% was reported to present positive impact for bone tissue engineering. β-TCP 20 wt% scaffolds provided strong evidence of enhanced long-term application. Moreover, the use of electrical stimulation as a non-invasive and complementary therapy boosted the bone regeneration effect of PCL/β-TCP scaffolds providing angiogenic and osteogenic stimuli, resulting in greater mineralized tissue formation [19,20,21]. Advancing and optimizing what was reported before, this study aimed to evaluate the in vivo use of composite scaffolds of polycaprolactone (PCL) mixed with hydroxyapatite and tricalcium phosphate, both ceramics and PCL mixed together (HA/TCP) and the ES therapy for the regeneration of critical size bone defects and the corresponding effects on the Wnt canonical and non-canonical pathways.

## 2. Material and Methods

### 2.1. Scaffold Fabrication

PCL pellets (Perstorp Caprolactones, Cheshire, UK) were heated up to 90 °C and mixed with 10 wt% of HA nanoparticles (Sigma-Aldrich, St. Louis, MO, USA, EUA) and 10 wt% of TCP microparticles (Sigma-Aldrich, St. Louis, EUA) for 20 min to produce a mixture containing 80 wt% PCL, 10 wt% HA, and 10 wt% TCP. The prepared composite material was 3D printed with a screw-assist 3D Discovery (REGENHU, Villaz-Saint-Pierre, Switzerland), using the 0/90° lay-down pattern. Fabricated scaffolds were sterilized in 70% ethanol for 4 h and rinsed with phosphate buffer (PBS) solution to remove the residual ethanol. The prepared scaffolds were ready to be grafted in the bone defect at the day of surgery.

### 2.2. In Vivo Study

All the surgical and experimental procedures were approved by the ethical principles in animal research adopted by Hermínio Ometto Foundation’s Ethics Committee on Animal Use (CEUA 075/2017), according to experimental standards and biodiversity rights (NIH Publication 80–23, revised 1996 and Arouca Law-11, 794, 2008).

There were 76 Wistar rats randomly and equally divided into four experimental groups, as listed in Table 1. Each group was further equally divided into three subgroups, considering experimental periods of 30, 60 and 120 days.

### 2.3. Surgical Protocol and Electrical Stimulation

The animals were anesthetized by the intraperitoneal administration of a mixture of ketamine hydrochloride (30 mg/kg) and xylazine hydrochloride (10 mg/kg). Trichotomy was performed in the occipital region. A critical-sized bone defect with dimensions of 4 mm × 6 mm was created in the calvary bone under constant irrigation with physiological solution (0.9% NaCl) using an Osteo I tip (Piezo Helse, Helse Dental Technology, Santa Rosa do Viterbo, SP, Brazil) coupled with a dental ultrasound handpiece (Olsen, Palhoça, SC, Brazil).

The scaffolds were grafted and precisely fitted to the bone defect without the need for clamping or physical fixation. After scaffold implantation, the animals were sutured with nylon 5-0 sutures (Shalon Medical, Goiânia, Brazil), followed by intraperitoneal and oral analgesic treatments for 72 h. The animals were monitored by the researchers.

**Figure 1:**
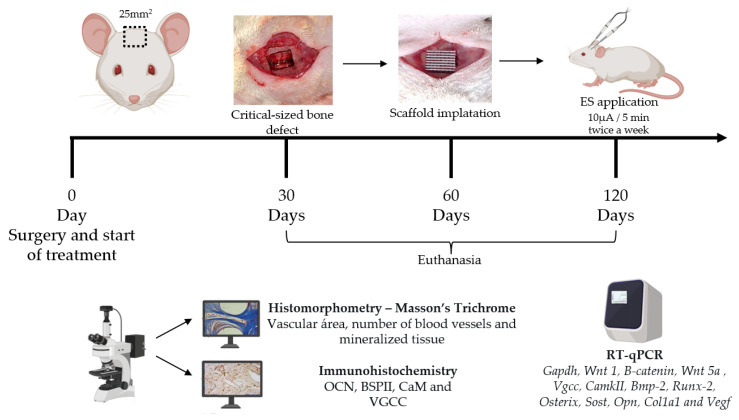
Scheme of the timeline of follow-up (surgery, treatments, euthanasia and sample harvesting) and experimental analysis.

The ES applications started immediately after surgical protocol and was applied twice a week throughout the three experimental periods (30, 60 and 120 days), using a low-intensity transcutaneous electrical stimulator (Physeotonus microcurrent, BIOSET, Indústria de Tecnologia Electrônica Ltd., Rio Claro, São Paulo, Brazil), for 5 min at 10 µA, as previously described [19,20,21]. The animals were euthanized after 30, 60, and 120 days of surgery with an anesthetic deepening and cervical dislocation. Figure 1 shows the timeline. Three samples from the bone defect area were collected for histomorphometric and immunohistochemical (n = 3/group/experimental period) evaluation, and were immediately fixed in 10% formaldehyde for 48 h. For the molecular evaluation, five samples were immediately frozen at −80 °C in 2 mL plastic tubes (n = 5/group/ experimental period).

### 2.4. Histomorphometry

After 48 h of fixation, the samples were demineralizing for 45 days, and the solution was changed three times per week. After demineralization, the samples were washed in water for 1 h, dehydrated in crescent ethanol concentration, diaphanized with xylol and embedded in paraffin. Cross-sections of 4.0 µm were mounted on glass slides and stained with Masson’s Trichrome (MT) for histomorphometric evaluation. The histological images were captured using the Leica DM2000 microscope (Leica Microsystems, Wetzlar, Germany) at 200× magnification to evaluate the number of blood vessels, vascular area, osteoid/collagen tissue, and mineralized tissue. Ten images of each bone sample were analyzed using ImageJ software (ImageJ 1.50b, Wayne Rasband, National Institutes of Health, Bethesda, MD, USA).

### 2.5. Immunohistochemistry

Histological cross-section samples were placed on silanized slides and incubated with the following primary antibodies (Table 2). The secondary antibodies and antibody detection reaction (NovolinkTM Max Polymer Detection System) were performed according to the manufacturer’s protocol (Leica Biosystems, Buffalo Grove, IL, USA) as previously reported [20,21]. Eight images of each bone sample were captured at 400× using the Leica DM2000 microscope (Leica Microsystems, Wetzlar, Germany) and quantified using ImageJ software (ImageJ 1.50b, Wayne Rasband, National Institutes of Health, USA).

### 2.6. Quantitative Polymerase Chain Reaction (qPCR)

Total RNA of the samples collected after 30- and 60-days post-surgery were macerated in liquid nitrogen and isolated using TRIzol^TM^ reagent (Invitrogen, Waltham, MA, USA) for RNA isolation, following the manufacturer’s instructions. The RNA was converted into cDNA from 1.5 µg of total RNA using the high-capacity kit (Thermo Fisher Scientific, Waltham, MA, USA) according to the manufacturer’s instructions. The TaqMan assays used are described in Table 3, were purchased from Applied Biosystems. The reactions were performed in triplicate with TaqMan Gene Expression Master Mix (Applied Biosystems, Waltham, MA, USA). The entire qPCR procedure was performed according to the protocol previously described [20,21]. The *Gapdh* was used as the control for data normalization and validated using the BestKeeper software. The PCL group was used as a calibrator and the results were calculated using the 2^−∆∆Ct^ method.

### 2.7. Statistical Analysis

All the experimental data are presented as the mean ± standard. Data were analyzed using GraphPad Prism 8 software (GraphPad Software, San Diego, CA, USA) and verified using the normality test. One-way ANOVA with Tukey’s post hoc test was applied for parametric data, while the Kruskal–Wallis test with Dunn post hoc was applied for non-parametric data. Significance levels were set at: * *p* < 0.05; ** *p* < 0.01; *** *p* < 0.001.

## 3. Results

### 3.1. Wnt Canonical Pathway

**Figure 2:**
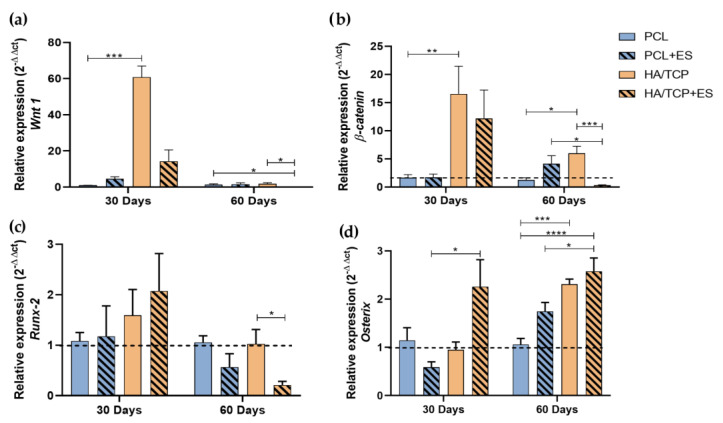
Relative expression of (**a**) *Wnt 1*, (**b**) *β-catenin*, (**c**) *Runx-2* and (**d**) *Osterix* (2^−ΔΔct^). Results were expressed as mean ± standard error of the mean (significance levels were established in * *p* < 0.05, ** *p* < 0.01, *** *p* < 0.001, **** *p* < 0.005).

To investigate the effects of HA/TCP composite scaffolds and ES application on bone regeneration, the Wnt pathway and osteogenic gene expression was assessed. The Wnt canonical pathway in the bone repair, promotes the expression of transcriptional factors such as *Runx-2* and *Osterix* that favor the osteoblastic differentiation. Figure 2 shows the gene expression of transcription factors *Wnt 1*, *β-catenin*, *Runx-2* and *Osterix*. At day 30, it can be observed that HA/TCP and HA/TCP + ES groups presented higher *Wnt 1* and *β-catenin* genes expression than those of PCL and PCL + ES. At day 60, the expressions of both *Wnt 1* and *β-catenin* presented a decreasing trend with the HA/TCP + ES group showing a significantly decrease. Moreover, the HA/TCP + ES group presented negative regulation when compared to the PCL + ES group.

As for *Runx-2* gene expression at day 30, results show no significant differences among the groups but the HA/TCP + ES group showed higher expression. After 60 days, all group exhibit a decrease trend in terms of *Runx-2* gene expression, particular for the HA/TCP + ES group.

Regarding the *Osterix* expression, it is possible to observe a greater expression in the HA/TCP + ES group compared to the others and significantly higher than PCL + ES group at day 30. After 60 days, all groups showed increased *Osterix* gene expression with that of HA/TCP + ES group significantly higher than those of PCL and PCL + ES groups. In addition, the HA/TCP group also presented higher expression than PCL group.

### 3.2. Wnt Non-Canonical Pathway: Wnt/Ca^2+^ and Ca^2+^/CaM Pathway

**Figure 3:**
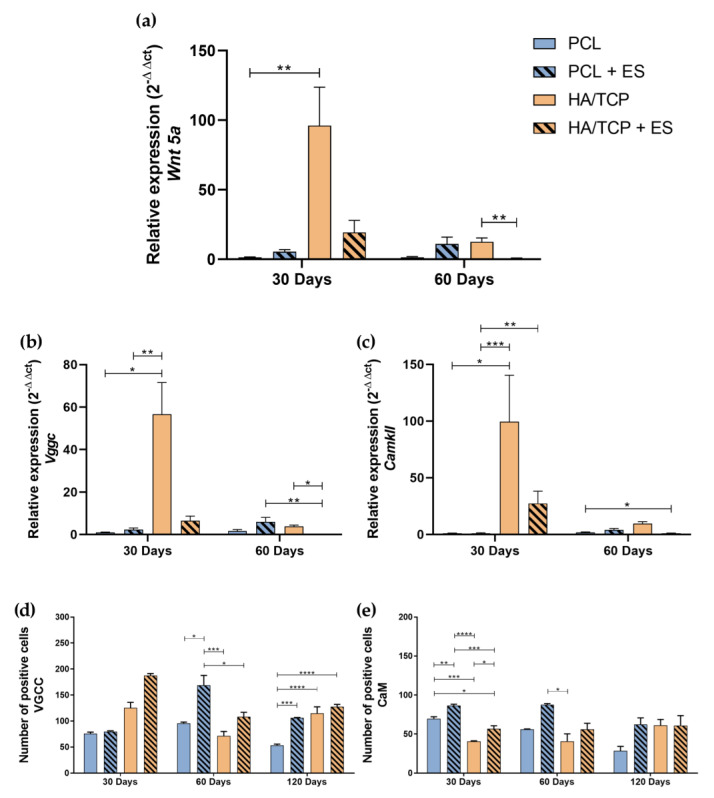
Relative gene expression of (**a**) *Wnt 5a*, (**b**) *Vgcc* and (**c**) *CamkII* (2^−ΔΔct^). Evaluation of the Ca^2+^/CaM signaling pathway by immunohistochemistry in 4 × 10^4^ μm^2^ area of images: (**d**) Quantification of anti-VGCC positive cells; (**e**) Quantification of anti-CaM positive cells. Results were expressed as mean ± standard error of the mean (significance levels were established in * *p* < 0.05, ** *p* < 0.01, *** *p* < 0.001, **** *p* < 0.005).

*Wnt 5a*, *CamkII*, *Vgcc* gene expression, and anti-VGCC, anti-CaM specific tissue markers (Figure S1) evaluations are shown in Figure 3. At day 30, it is possible to observe that the HA/TCP group showed significantly gene expressions of *Vgcc*, *CamkII* and *Wnt 5a* compared to PCL and PCL + ES groups (Figure 3a–c). Moreover, the HA/TCP + ES group also presented high expressions of *Vgcc*, *CamkII* and *Wnt 5a* compared to the PCL group. At Day 60, the HA/TCP + ES group presented lower gene expression, a negative regulation, for the same genes compared to the other groups.

Figure 3d shows that, although no statistically significant differences were observed, the HA/TCP and HA/TCP + ES groups presented a greater cellularity expression of VGCC marker, especially the HA/TCP + ES group. However, after 60 days, the PCL + ES group presented a statistical higher VGCC positive cell number than other groups and after 120 days, PCL + ES, HA/TCP, and HA/TCP + ES all presented higher cellularity compared to the PCL group. In terms of CaM positive cells, it can be seen in Figure 3e that PCL + ES group presented higher CaM positive cells than the others and PCL group showed greater number than HA/TCP and HA/TCP + ES groups, and HA/TCP + ES greater than HA/TCP after 30 days. Interestingly, at 60 and 120 days, CaM positive cells followed the same trend as VGCC for all groups.

### 3.3. Osteogenesis

**Figure 4:**
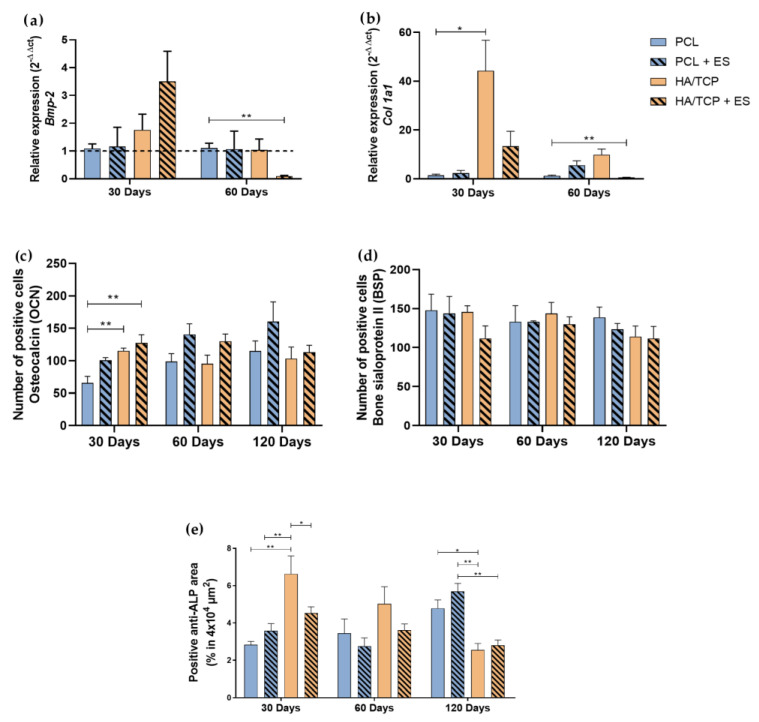
Relative expression of (**a**) *Bmp-2*, (**b**) *Col1a1* (2^−ΔΔct^). Evaluation of BSPII, OCN and ALP by immunohistochemistry in 4 × 10^4^ μm^2^ area of images: (**c**) Quantification of anti-OCN positive cells, (**d**) quantification of anti-BSPII positive cells, (**e**) quantification of anti-ALP area. Results were expressed as mean ± standard error of the mean (significance levels were established in * *p* < 0.05, ** *p* < 0.01).

The BMP-2 is an important osteogenic marker that actuates like a growth factor that can induce the expression of non-collagen proteins such as osteocalcin (OCN), bone sialoprotein II (BSPII) and some osteoblastic differentiation markers such as alkaline phosphatase (ALP) and collagen type I (*Col1a1*). The expression of these markers is essential for the mineralization process (Figure S1). To investigate the osteogenesis behavior of scaffolds and the effect of ES, *Bmp-2* and *Col1a1* gene expression, anti-OCN and anti-BSPII number of positive cells, and alkaline phosphatase (ALP) positive area (specific tissue markers) were performed as shown in Figure 4. Results showed that the HA/TCP + ES group presented higher *Bmp-2* expression after 30 days without significant differences while a significant decrease was observed for the HA/TCP + ES group at day 60. A high *Col1a1* expression was observe at day 30 for the HA/TCP and HA/TCP + ES groups. However, only the HA/TCP group was statistically different from the PCL group. After 60 days, the HA/TCP and HA/TCP + ES groups showed a decreasing trend, particularly for the HA/TCP + ES group which was significantly lower than the PCL group.

As shown in Figure 4c, the HA/TCP and HA/TCP + ES groups exhibit a higher number of OCN positive cells compared to the PCL group at day 30. At day 60, the groups treated with ES showed higher number of OCN positive cells than those without ES. Similar trend can be also observed at day 120 with the PCL + ES showing the highest value compared to other groups. As for the BSP positive cell test (Figure 4d), there is no significant difference between each group and the number of BSP positive cells were slightly decreasing at days 60 and 120. The HA/TCP group presented statistically higher percentage of ALP area than the other groups, especially compared to the PCL group but showed a decreasing trend at days 60 and 120 while the PCL and PCL + ES group increased along with time. At 120 days, the HA/TCP and HA/TCP + ES groups presented a statistically smaller percentage of ALP area than PCL and PCL + ES groups. 

**Figure 5:**
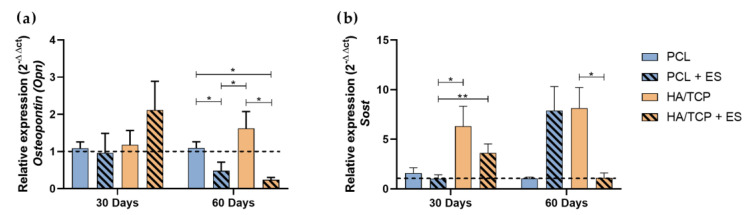
Relative gene expression of (**a**) *Osteopontin (Opn)* and (**b**) *Sost* (2^−ΔΔct^). Results were expressed as mean ± standard error of the mean (significance levels were established in * *p* < 0.05, ** *p* < 0.01).

Some proteins are expressed after the mineralization process. Osteopontin acts regulating the bone matrix mineralization process, while Sost is a protein expressed by osteocytes and plays an important role as an inhibitor of the Wnt canonical pathway, preventing osteoblastic differentiation. The relative gene expression of *Osteopotin (Opn)* and *Sost* is shown in the Figure 5. At day 30, no significant statistical differences were observed between each group regarding the expression of *Opn* but the HA/TCP + ES group presented a higher expression. At day 60, the PCL group presented a higher expression compared to the PCL + ES and HA/TCP + ES groups and the HA/TCP group showed a higher expression than the HA/TCP + ES and PCL + ES groups.

Regarding the *Sost* expression, the HA/TCP and HA/TCP + ES groups presented higher expressions than PCL and especially the PCL + ES group at day 30. After 60 days, PCL and HA/TCP + ES group presented lower expression compared to PCL + ES HA/TCP group.

### 3.4. Angiogenesis and Mineralization

**Figure 6:**
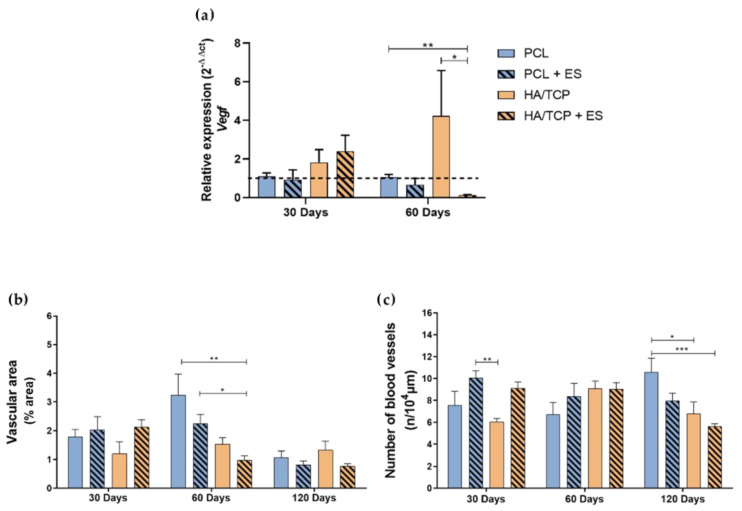
Evaluation of angiogenesis by (**a**) relative expression of *Vegf* (2^−ΔΔct^); (**b**) vascular area and (**c**) number of blood vessels formed by imaging. Results were expressed as mean ± standard error of the mean (significance levels were established in * *p* < 0.05, ** *p* < 0.01, *** *p* < 0.001).

Figure 6 shows the relative gene expression of *Vegf*, the vascular area and the number of blood vessels at different time points. Results showed that there was no evident difference between each group in terms of the *Vegf* expression at day 30. However, the HA/TCP + ES and HA/TCP groups demonstrated a higher expression than non-bioceramic groups. After 60 days, the HA/TCP + ES group showed lower expression of *Vegf* compared to the other groups and the HA/TCP still presented the highest value of *Vegf* gene expression. As for the vascular area, it can be seen that the area decreases by increasing the time and no significant differences were observed among the different groups at days 30 and 120. However, the HA/TCP + ES group exhibited the lowest vascular area compared to both the PCL and PCL + ES groups. Regarding the number of vessels, the PCL + ES group showed a higher number than the HA/TCP group after 30 days and the HA/TCP and HA/TCP + ES groups showed statistical lower values than the PCL group at day 120.

**Figure 7:**
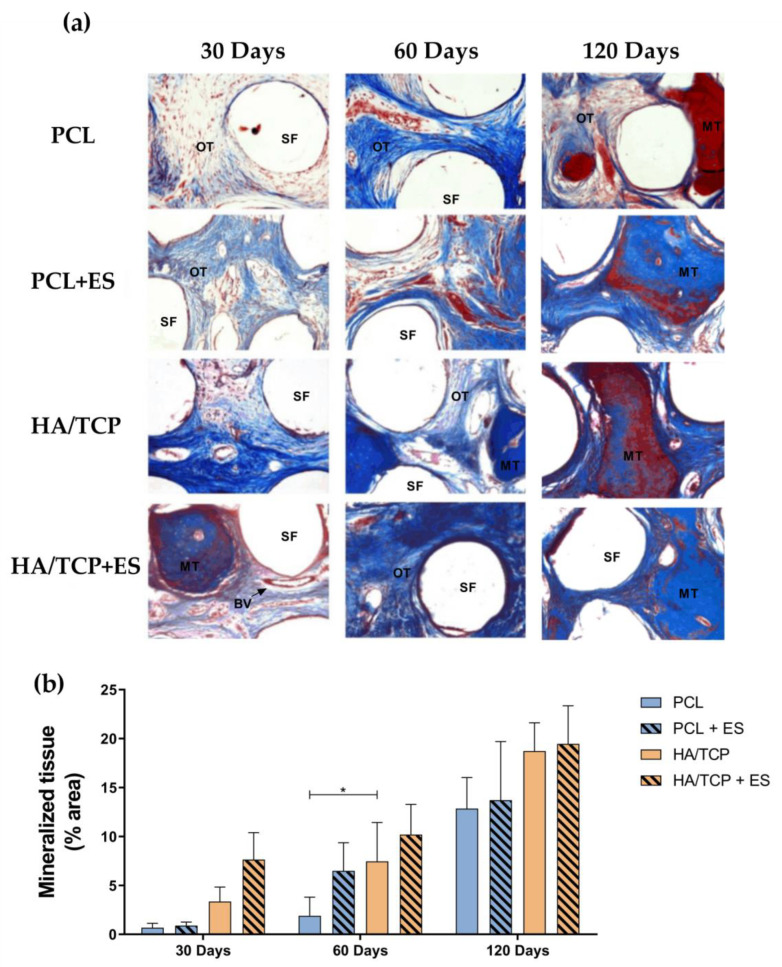
(**a**) Qualitative histological evaluation of the experimental groups PCL, PCL + ES, HA/TCP and HA/TCP + ES. SF: scaffold filament, OT: osteoid tissue, MT: mineralized tissue, BV: blood vessel. Photomicrograph at 200× magnification of the defect area after 30, 60 and 120 days of the surgical procedure stained with Masson’s Trichrome. Evaluation of the bone formation process: (**b**) percentage of mineralized tissue in all groups after 30 and 60 days. Images stained with Masson’s trichrome at 200× magnification were used for quantification. Results were expressed as mean ± standard error of the mean (significance levels were established in * *p* < 0.05).

Figure 7 represents the histological images of the Masson’s trichrome stained bone defect area treated with different scaffolds as well as the semi-quantification of the percentage of mineralized tissue at days 30, 60 and 120. As observed from Figure 7a, HA/TCP and HA/TCP + ES groups presented compact and dense bone at day 30 and this trend was strengthened along with the implantation time. The quantification of bone formation is shown in Figure 7b. As illustrated in Figure 7a, since day 30, HA/TCP and HA/TCP + ES groups showed greater mineralization than PCL and PCL + ES groups. Similar trend was also observed at days 60 and 120 with a constant increase in mineralized tissue, particularly in the HA/TCP and HA/TCP + ES groups.

## 4. Discussion

Bone regeneration strongly depends on two fundamental events: osteogenesis and effective mineralization, which is also highly associated with adequate angiogenesis [22,23,24]. This process can be modulated by using ES which stimulates the VEGF production or using phosphates and calcium, which was also endowed with the angiogenic potential [25,26]. In this study, although no significant differences were observed regarding the expression of *Vegf* at day 30, results indicate an increase in both the number of blood vessels and vascular area in the groups treated with the ES. These results are aligned with the findings of Leppik et al. [27], who observed an increase in vascular growth after the application of ES. However, as expected in bone regeneration, the overall reduction in vascularization area for all the other groups is directly associated with the tissue maturation and mineralization, which corresponds to a physiological reduction in blood vessels. The quicker reduction in angiogenesis in HA/TCP and HA/TCP + ES groups observed at day 60 for the vascular area, and day 120 for the number of blood vessels is inversely proportional to the mineralization.

While the osteoinductive and osteoconductive properties of the biomaterial in association with ES contributed to the angiogenic process, this combination can also contribute to the increase in the expression of fundamental genes for cell differentiation, such as *Bmp-2*, *Runx-2* and *Osterix* [15,27,28]. The high expression of *Bmp-2, Runx-2* and *Osterix* in the HA/TCP + ES group at day 30 confirms this hypothesis. 

However, beyond inducing *Vegf* expression in osteoblasts, the high expression of *Bmp-2* at day 30 may have contributed to the late expression of *Osterix* after the 60th day in the HA/TCP + ES group. This event may be due to the positive regulation of *Bmp-2* after the ES application [27], since *Bmp-2* contributes to osteogenic differentiation, and to bone tissue formation and mineralization. *Runx-2* and *Osterix* are transcriptional factors expressed during the cell differentiation process. *Runx-2* is initially expressed by osteoprogenitor cells and pre-osteoblasts, while *Osterix* is expressed by mature osteoblasts [21].

The activation of the Wnt/β-catenin pathway, also known as the canonical Wnt pathway, can promote the expression of *Runx-2* and *Osterix*. Wang et al. [28] observed that scaffolds composed of HA/TCP can stimulate the canonical Wnt pathway, corroborating the *Wnt 1* and *β-catenin* expression profile of the HA/TCP group at days 30 and 60 observed in this study. Other authors stated that the application of ES is able to modulate the expression levels of genes such as *Wnt 1* and *β-catenin* [29,30]. However, our results showed low expressions of *Wnt 1* and *β-catenin* in the HA/TCP + ES group at days 30 and 60. This can be attributed to an accelerated differentiation and mineralization process before day 30. This hypothesis arises from the histological results that showed greater mineralization and reduced vascularization in the HA/TCP + ES group throughout the experimental period. Moreover, it was reported that the expression of genes related to both the canonical and non-canonical Wnt pathways might have the peak expression within 14 days after injury and return to the basal expression level between 14 and 21 days [31]. Shorter experimental periods will be further conducted by the authors to corroborate this.

Regarding the findings related to the non-canonical Wnt/Ca^2+^ pathway, the expression of *Wnt 5a* was higher in the HA/TCP compared to the other groups. According to Schupbach et al. [29], it is suggested that the lower expression of *Wnt 5a* in animals treated with ES (HA/TCP + ES) may be due to the accelerated mineralization process, since this ligand is superiorly expressed at the injured site. By binding to its receptor, it plays a fundamental role in osteogenic differentiation, increasing the expression of *Bmp-2*. Some studies suggested that bone cell proliferation by using ES may be mediated by the calcium influx by voltage gated calcium channel (VGCC), which is responsible for activating the Ca^2+^/CaM pathway [17,32]. Although *Vgcc* and *CamkII* presented low gene expressions in HA/TCP + ES group, our data showed greater VGCC labeling by immunohistochemistry for both ES-stimulated groups. Results suggested gene expression earlier than the 30th day, and the activation of calcium channels and calmodulin pathway due to the ES therapy.

Similar findings were observed by Bagne et al. [17], showing that the groups treated with ES exhibited an increase in the expression of *Vgcc*. The activation of this type of channel promotes an increase in the amount of cytosolic calcium, responsible for the activation of calmodulin, resulting in an increase in cell proliferation [31]. Our findings also showed that the CaM labeling was higher, both at 30 and 60 days in the groups that received the application of ES, also corroborating previously reported results [17].

The high expressions of *Runx-2* in HA/TCP and HA/TCP + ES groups are good biological indications of the proliferative phase that pre-osteoblasts begin to express, producing ALP, *Col1a1* and BSPII [33,34,35]. These results justify the higher labeling of ALP and BSPII in the HA/TCP group at 30 and 60 days and the high expression of *Col1a1* at 30 days in the same group. However, the lower expression of *Col1a1* at 30 days, the lower labeling of ALP and BSPII (both at 30 and 60 days) and the drop in ALP labeling in the HA/TCP and HA/TCP + ES groups at 120 days can be attributed to the high expression of *Osterix* in these groups, which is particularly relevant in the HA/TCP + ES group at 30 and 60 days [34]. The increase in *Osterix* expression reduces the expression of early markers of osteoblastic differentiation such as *Col1a1*, ALP and BSPII, favoring the synthesis of other proteins related to mineralization such as OCN [32,33]. It is known that OCN has a higher expression in immature osteocytes that are close to the bone formation area, especially in mineralized regions [34]. Our findings showed that the HA/TCP + ES group had a higher number of OCN positive cells in 30 days, corroborating the amount of mineralized tissue observed in this group.

Mineralization is considered a hallmark of the end of osteoblast differentiation. Puttini et al. [2] observed, from histological results, mineralized tissue formation using HA/TCP scaffolds (60 wt%/40 wt%) which presented bone formation after 30 days of implantation and greater bone formation after 60 days of evaluation, corroborating with our results. In addition, HA/TCP scaffolds at two different concentrations showed greater bone formation at 60 days in the calvaria of rabbits, when compared to the group not receiving any scaffold [36,37]. Finally, Schmidlin et al. [38] also observed greater mineralization at 30 days in the groups that received HA/TCP scaffolds when compared to β-TCP. Although the scaffolds were produced using different techniques than those used in this work, it is possible to observe the osteoinductive and osteoconductive potential of this material.

The bone formation process is also characterized by the expression of some specific markers such as *Osteopontin* and *Sost* [39,40]. Srirussamee et al. [22] reported higher levels of *Osteopontin* expression after the application of ES, confirming that its use accelerates the mineralization and maturation process [38]. In turn, Poh et al. [41] suggested that scaffolds composed of CaPs such as HA and TCP may help in osteoblastic differentiation, contributing to an increase in *Osteopontin* expression.

Furthermore, although the HA/TCP + ES group showed the greater mineralization, the increase in *Sost* expression in this group and especially in the HA/TCP group is an indication of modulation of bone deposition, as this gene is involved in the promotion of osteoblast apoptosis, inhibiting their conversion by BMPs, thus blocking bone formation [17]. However, according to Schupbach et al. [29], *Sost* expression is reduced at the beginning of the repair process when compared to a bone without injury, presenting a slight increase after 7 days of injury and returning to a level below an intact bone between 14 and 21 days, which may explain the lower expression observed in the HA/TCP + ES group.

## 5. Conclusions

The results presented in this paper show that the use of composite scaffolds of PCL and 10 wt% of HA and 10 wt% of TCP with and without application of ES exhibited better results regarding bone repair, greater stimuli to the expression of bone markers and Wnt genes, especially at early experimental time (30 days), obtaining greater mineralization when compared to the PCL group. Moreover, the use of electrical stimulation boosted the bone regeneration effect. The application of ES allowed fast angiogenesis in the initial period and positively influenced the expression of bone markers in the early stages (30 days) of bone maturation. Greater and faster mineralized tissue is related to Wnt pathway, especially the Ca^2+^/CaM pathway.

## Figures and Tables

**Table 1:** Experimental groups for in vivo study.

Group Name	ES	PCL Concentration	Ceramics Concentration
PCL	No	100 wt%	0 wt%
PCL + ES	Yes	100 wt%	0 wt%
HA/TCP	No	80 wt%	HA 10 wt% + TCP 10 wt%
HA/TCP + ES	Yes	80 wt%	HA 10 wt% + TCP 10 wt%

**Table 2:** Antibodies used for immunostaining.

Antibody	Dilution	Codes	Company
Ostecalcin (OCN)	1:50	sc-365797	Santa Cruz Biotechnology, Dallas, TX, USA
Bone Sialoprotein II (BSP-II)	1:250	sc-73630	
Calmodulin (CaM)	1:200	sc-137079	
Voltage-Gated Calcium Channel (VGCC)	1:100	sc-515679	

**Table 3:** TaqMan assays used for RT-qPCR.

TaqMan Assays	Product Codes
*Gapdh* Glyceraldehyde-3-Phosphate Dehydrogenase	Rn01775763_g1
*Vegf* Vascular endothelial growth factor	Rn01511602_m1
*Runx-2* Runt-related transcription factor 2	Rn01512298_m1
*Osterix* Transcription factor Sp7	Rn02769744_s1
*Bmp-2* Bone morphogenetic protein-2	Rn00567818_m1
*Opn* Osteopontin	Rn00681031_m1
*Sost* Sclerostin	Rn00577971_m1
*Col 1α1* Collagen type I, alpha 1	Rn01463848_m1
*Wnt 1* Wnt family member 1	Rn01761722_m1
*β-catenin*	Rn00584431_g1
*Wnt 5a* Wnt family member 5a	Rn01402000_m1
*CamkII* Calmodulin-dependent protein kinase II	Rn00572627_m1
*L-type* Voltage-Gated Calcium Channel	Rn01453395_m1

## Data Availability

Data available on request from the corresponding author due to privacy and also ethical issues.

## Supplementary Materials

The following supporting information can be downloaded at: https://www.mdpi.com/article/10.3390/bioengineering10010075/s1, Figure S1. Immunohistochemistry images.
