# Supplementary figures and images for: Electrical Stimulation Therapy and HA/TCP Composite Scaffolds Modulate the Wnt Pathways in Bone Regeneration of Critical-Sized Defects
Source: Bioengineering (Basel). 2023 Jan 6;10(1):75. doi: 10.3390/bioengineering10010075 (PMC9854456; doi:10.3390/bioengineering10010075)

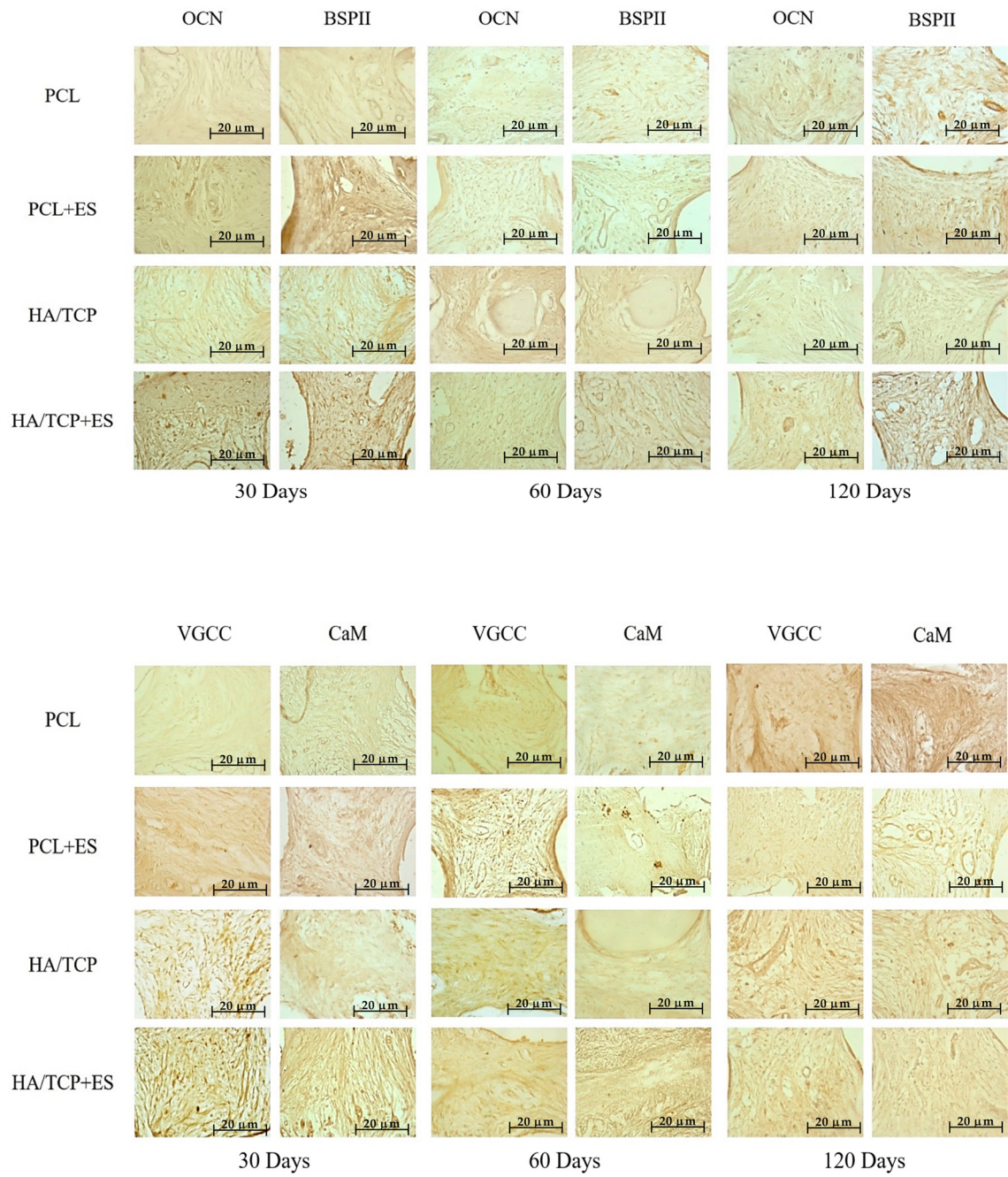

Figure S1. Immunohistochemistry images.

Supplement: Supplementary file 1 [file bioengineering-10-00075-s001.zip › bioengineering-2038951-supplementary.pdf]
